# Differential Responses of Antioxidant Enzymes and Lignin Metabolism in Susceptible and Resistant Sweetpotato Cultivars during Root-Knot Nematode Infection

**DOI:** 10.3390/antiox12061164

**Published:** 2023-05-27

**Authors:** Jung-Wook Yang, Sul-U Park, Hyeong-Un Lee, Ki Jung Nam, Kang-Lok Lee, Jeung Joo Lee, Ju Hwan Kim, Sang-Soo Kwak, Ho Soo Kim, Yun-Hee Kim

**Affiliations:** 1 Department of Crop Cultivation & Environment, Research National Institute of Crop Science, Rural Development Administration, Suwon 16200, Republic of Korea; 2 Plant Systems Engineering Research Center, Korea Research Institute of Bioscience and Biotechnology (KRIBB), Daejeon 34030, Republic of Korea; 3 Bioenergy Crop Research Institute, National Institute of Crop Science, RDA, Muan 58538, Republic of Korea; 4 Department of Biology Education, IALS, Gyeongsang National University, Jinju 52609, Republic of Korea; 5 Department of Plant Medicine, IALS, Gyeongsang National University, Jinju 52609, Republic of Korea; 6 Department of Pharmacology, College of Medicine, Dankook University, Cheonan 31008, Republic of Korea

**Keywords:** antioxidant enzyme, lignin metabolism, resistant cultivars, root-knot nematode, susceptible cultivars, sweetpotato

## Abstract

Root-knot nematodes (RKN) cause significant damage to sweetpotato plants and cause significant losses in yield and quality. Reactive oxygen species (ROS) play an important role in plant defenses, with levels of ROS-detoxifying antioxidant enzymes tightly regulated during pathogen infection. In this study, ROS metabolism was examined in three RKN-resistant and three RKN-susceptible sweetpotato cultivars. The antioxidant enzymes superoxide dismutase (SOD), catalase (CAT), and peroxidase (POD) were assessed, as was lignin-related metabolism. In RKN-infected roots, both resistant and susceptible cultivars increased SOD activity to produce higher levels of hydrogen peroxide (H_2_O_2_). However, H_2_O_2_ removal by CAT activity differed between cultivars, with susceptible cultivars having higher CAT activity and lower overall H_2_O_2_ levels. In addition, the expression of phenylpropanoid-related phenylalanine ammonia-lyase and cinnamyl alcohol dehydrogenase genes, which encode enzymes involved in lignin metabolism, were higher in resistant cultivars, as were total phenolic and lignin contents. Enzyme activities and H_2_O_2_ levels were examined during the early (7 days) and late (28 days) phases of infection in representative susceptible and resistant cultivars, revealing contrasting changes in ROS levels and antioxidant responses in the different stages of infection. This study suggests that differences in antioxidant enzyme activities and ROS regulation in resistant and susceptible cultivars might explain reduced RKN infection in resistant cultivars, resulting in smaller RKN populations and overall higher resistance to infection and infestation by RKNs.

## 1. Introduction

Root-knot nematodes (RKNs) (*Meloidogyne* spp.) are obligate endoparasites that infect a variety of plant species and are among the most damaging crop parasites worldwide [1]. RKNs parasitize plant root systems, impacting the absorption of water and nutrients and leading to effects at the whole-plant level. Crop yields are then reduced, resulting in significant global economic losses of millions of dollars [2]. RKN infection occurs when motile larvae are attracted to the root system by root exudates and enter the elongation zone just behind the root tip [3]. Feeding sites in the plant are characterized by the presence of hypertrophic multinucleated cells (giant cells) produced by successive mitotic divisions occurring without cytokinesis. During development, RKNs use stylets to feed on these cells and obtain nutrients from the host plant, resulting in an abnormal distribution of photosynthesis at the feeding site. This damage leads to wilting, stunted growth, and increased susceptibility to plant pathogens. Some varieties of usually susceptible plant species display natural resistance to attack by RKNs. Understanding the genetic, molecular, and biochemical factors underpinning natural resilience to RKN infection is of great interest for the development of resistant cultivars.

Sweetpotato (*Ipomoea batatas* Lam.), a member of the *Convolvulaceae* family, is a widely cultivated root crop species. Sweetpotato roots have a range of uses, including as food for humans and animals as well as in industrial processes such as alcohol, starch, and natural pigment production. Sweetpotato exhibits antioxidant activity and can thus be designated as a functional food [4,5]. The RKN *Meloidogyne incognita* causes considerable damage to sweetpotato plants and causes significant losses in yield and quality [6,7]. Some nematicides are effective against RKNs but can be harmful to human health, soil, and the environment. Developing plant cultivars with resistance to RKN is thus a desirable strategy for the management of RKNs. Plants exhibit a range of biochemical and physical responses in response to RKN infection, including the production of compounds that are toxic to RKNs. Plant resistance to RKN is also characterized by the hypersensitivity response (HR), which rapidly induces localized cell death in infected plants.

The primary and immediate response of plants to pathogens is the overproduction of reactive oxygen species (ROS) at the site of infection, such as superoxide anion (O_2_^•−^) and hydrogen peroxide (H_2_O_2_). These ROS then induce defense reactions, including HR. Precise regulation of ROS production and elimination in plant tissues is critical to avoiding irreversible cell damage that can be caused by excessive ROS [8]. Several enzymes work together to tightly regulate the plant antioxidant network to maintain steady-state levels of ROS in plant cells [9]. For example, superoxide dismutase (SOD) catalyzes the decomposition of superoxide anions into H_2_O_2_, whereas catalase (CAT) as the main antioxidant enzyme in peroxisome catalyzes the decomposition of H_2_O_2_, generating H_2_O and oxygen [10]. Guaiacol peroxidase (POD) catalyzes the H_2_O_2_-dependent polymerization of hydroxy cinnamyl alcohol during lignin biosynthesis and the H_2_O_2_-dependent strengthening of cell wall proteins such as hydroxyproline-rich glycoproteins [11].

Nematode resistance genes have been identified in several plant species, most of which are associated with the typical HR response of root cells to invasive nematodes. Many biochemical and histochemical studies have suggested the concomitant activation of enzymes involved in ROS metabolism [12,13]. One common response to RKN attack in host plants harboring resistance genes is early HR-mediated cell death surrounding the nematode feeding site, which prevents the nematode from feeding further and results in nematode death. HR symptoms and H_2_O_2_ production have also been reported from incompatible interactions between *Arabidopsis* and the cyst nematode (CN) *Heterodera glycines* [14]. One of the best-characterized nematode resistance genes is *Mi*, which confers resistance to three RKN species in tomato [15]. In addition, strong early HR responses were associated with *Mex-1*-mediated resistance in coffee [16], *Me3*-mediated resistance in black pepper [17], and soybean incompatibility interactions [18]. Responses occurring in resistant tomatoes infected with avirulent RKN were associated with additional molecular changes, including the regulation of enzymatic activities involved in the generation and scavenging of ROS [19,20]. However, despite some fragmentary data, the generation and precise function of ROS during nematode infection and the roles of ROS-mediated plant defense responses in the interaction of nematodes with individual plants have only been fully explored in a few plant species.

Our previous research examined representative RKN-susceptible and RKN-resistant sweetpotato cultivars using proteome and transcriptome analysis [21,22]. This was followed by the comparative transcriptome profiling of three RKN-resistant cultivars (RCs) with three RKN-susceptible cultivars (SCs) [23]. The expression levels of several candidate genes thought to be involved in RKN resistance correlated with the comparative transcriptomic analysis of the RCs and SCs. However, although this enabled the classification of sweetpotato cultivars as either resistant or susceptible to RKN infection, the roles and mechanisms of ROS-mediated defense responses in RKN resistance remained unclear. Therefore, this study aimed to investigate changes in ROS metabolism during RKN infection in susceptible RCs and to investigate ROS-mediated defense responses against nematode resistance during early and late infection. In this study, enzyme activity assays and gene expression analysis were used to explore the antioxidant defense metabolic pathways involved in ROS regulation during RKN infection. This is the first reported study of the ROS-mediated resistance response in nematode RCs and SCs in sweetpotato.

## 2. Materials and Methods

### 2.1. Plant Materials

Six sweetpotato (*Ipomoea batatas L. Lam)* cultivars were obtained from the Bioenergy Crop Research Center, National Crop Research Institute (RDA, Muan, Jeonnam, Republic of Korea). Three were RKN-SCs (DHM: Dahomi, SHM: Shinhwangmi, and YM: Yulmi) and three were RKN-RCs (DJM: Danjami, PWM: Pungwonmi, and JHM: Juhwangmi). The resistance characteristics of the six cultivars were explored in previous research [23].

### 2.2. Plant Treatment with M. incognita

Sweetpotato plants were infected with *M. incognita* as described previously [23]. Fifteen plants per cultivar were planted in perforated 500 cm^3^ clay pots in a sterile sand:soil mixture (50:50). The pots were randomly arranged and maintained in a greenhouse at 25–30 °C for 2 weeks, after which, approximately 3000 *M. incognita* eggs were applied to the soil of each pot and covered with a layer of moist sand. Roots were harvested 4 weeks after inoculation and stained with 0.015% Phloxin B solution for 15 min for visual assessment of gall numbers on roots [24]. Additional sweetpotato root samples were ground in liquid nitrogen and stored at −70 °C until required for further experiments.

### 2.3. Analysis of H_2_O_2_ Contents

The H_2_O_2_ content of sweetpotato roots was evaluated using the xylenol orange method as previously described [25]. Total H_2_O_2_ content was expressed as µmol of H_2_O_2_ per gram of dry weight of root tissue at 560 nm using an extinction coefficient of 43.6 M^−1^cm^−1^.

### 2.4. Enzyme Activity Assays

Total soluble protein was extracted from RKN-infected roots of sweetpotato plants using extraction buffer, and the concentration of total protein was determined using a Bradford assay with Bio-Rad reagent [26]. Extracted proteins were used for the determination of specific SOD, CAT, and POD activities. For the SOD activity assay, a fine powder of sweetpotato root was resuspended in 50 mM potassium phosphate (pH 7.8) with 0.1 mM EDTA, and then centrifuged at 12,000× *g* for 15 min at 4 °C. SOD activity was assayed using the photochemical nitro blue tetrazolium (NBT) method [27]. The photoreduction of NBT (formation of purple formazan) was measured at 560 nm, and inhibition curves were constructed for different volumes of protein extract. One unit of SOD is defined as being present in the extract volume that inhibits 50% of NBT photoreduction. For the CAT activity assay, frozen sweetpotato roots were ground in liquid nitrogen and homogenized in 0.1 M potassium phosphate buffer (pH 7.0). CAT activity was assessed in the supernatant after 15 min of centrifugation at 12,000× *g* at 4 °C, according to previously described methods [28]. CAT activity was determined by the decrease in absorbance at 240 nm over 1 min due to H_2_O_2_ consumption using an extinction coefficient of 39.4 mM^−1^cm^−1^. For POD activity assays, frozen sweetpotato roots were ground in liquid nitrogen and homogenized in 0.1 M potassium phosphate buffer (pH 6.0). POD activity was assessed in the supernatant after 15 min of centrifugation at 12,000× *g* at 4 °C, using pyrogallol substrate, according to previously described methods [29]. One unit of POD activity was defined as the amount of enzyme required to form 1 mg of purpurogallin from pyrogallol in 20 s, as measured by absorbance at 420 nm using an extinction coefficient of 2.47 mM^−1^cm^−1^.

### 2.5. Determination of Total Phenolic and Lignin Contents

Soluble phenols and insoluble lignin were extracted in methanol and assessed as described previously [30]. The methanol extract was centrifuged at 12,000× *g* for 10 min, and the supernatant was used for Folin–Ciocalteau analysis. For the analysis of total phenolics, soluble phenol was determined spectroscopically at 725 nm with a molar extinction coefficient of 5.228 ± 0.187 × 103 M^−1^ cm^−1^ using p-coumaric acid as a standard. For the analysis of total lignin, the remaining root tissue was dried at 60 °C for 48 h and powdered before the quantification of lignin content using a thioglycolic acid assay [30,31]. Briefly, 50 mg dry powder was treated with 0.5 mL of a 1:10 mixture of thioglycolic acid and 2N HCl at 100 °C for 4 h. After two consecutive water washes, lignothioglycolic acid was extracted from the pellets with 1 mL of 0.5N NaOH for 18 h. The lignin content was measured at 280 nm with an extinction coefficient of 22.9 L g^−1^ cm^−1^ using a lignin standard (alkali, 2-hydroxypropylether, Aldrich, St Louis, MO, USA).

### 2.6. RNA Extraction and qRT-PCR Analysis

Total RNA was isolated from sweetpotato roots using a Trizol RNA isolation kit (Invitrogen, Carlsbad, CA, USA). Quantitative real-time (qRT)-PCR analysis was performed on a Bio-Rad CFX96 thermal cycler (Bio-Rad, Hercules, CA, USA) using EvaGreen fluorescent dye according to the manufacturer’s instructions. Data were normalized relative to the mean CT value of the reference gene ubiquitin extension protein (UBI) and ADP-ribosylation factor (ARF) [32]. The gene-specific PCR primer sets used in this study are listed in Table S1.

### 2.7. Statistical Analysis

Data were analyzed using one-way analysis of variance (ANOVA). The statistical significance level was set at *p* < 0.05. Subsequent multiple comparisons were examined using Tukey’s multiple range test. All statistical analyses were performed using the Statistical Package Software for Social Sciences (SPSS ver. 29).

## 3. Results

### 3.1. Identification and H_2_O_2_ Analysis of RKN-Resistant and -Susceptible Sweetpotato Cultivars

**Figure 1:**
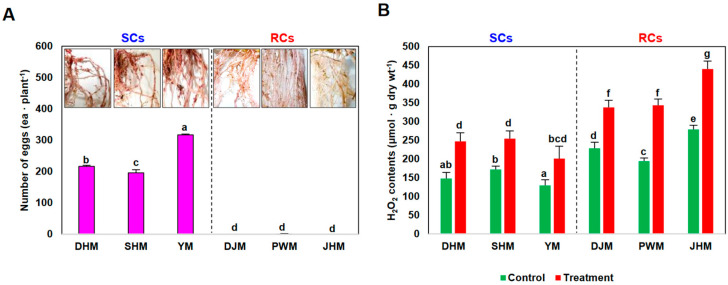
Effect of the root-knot nematode (RKN) *Meloidogyne incognita* on susceptible and resistant sweetpotato cultivars. (**A**) Numbers of egg masses in sweetpotato roots 4 weeks after RKN treatment. (**B**) H_2_O_2_ contents in sweetpotato roots 7 days after RKN treatment. Bars denoted with the same letter are not significantly different (*p* = 0.05) according to Tukey’s test. SCs, susceptible cultivars; RCs, resistant cultivars; DHM, Dahomi; SHM, Shinhwangmi; YM, Yulmi; DJM, Danjami; PWM, Pungwonmi; and JHM, Juhwangmi.

The RKN-RCs (resistant cultivars: Danjami (DJM), Pungwonmi (PWM), and Juhwangmi (JHM)), RKN-SCs (susceptible cultivars: Dahomi (DHM), Shinhwangmi (SHM), and Yulmi (YM)) used in this study were previously shown to exhibit high and low resistance to RKN infection, respectively [23]. Therefore, in this study, the resistance levels of SCs and RCs were first assessed against *M. incognita* by measuring the formation of nematode egg masses after infection with RKN in greenhouse conditions (Figure 1A). Roots were assessed 1 week (7 days) and 4 weeks (28 days) after RKN egg infection. Regardless of cultivar, roots were not significantly altered at 1 week after RKN infection. RKN eggs were found 4 weeks after infection, but only in SCs. *M. incognita* formed 216, 195, and 316 egg masses on SCs DHM, SHM, and YM plants, respectively, but only 0–1 egg masses on RCs DJM, PWM, and JHM (Figure 1). These results are consistent with previous studies and confirm the substantial difference in the level of *M. incognita* resistance between SCs and RCs [23]. Hydrogen peroxide (H_2_O_2_), an ROS, suppresses disease through its mediation of the hypersensitive (HR) response. In this study, H_2_O_2_ levels were compared after infection with *M. incognita* in three sweetpotato RCs (DJM, PWM, and JHM) and three SCs (DHM, SHM, and YM) (Figure 1B). During the early infection stage, 1 week after treatment with RKNs, the H_2_O_2_ levels were elevated in all six cultivars. Higher H_2_O_2_ levels were observed in the three RCs than in the three SCs, in both untreated and RKN-treated roots. The three RCs exhibited higher H_2_O_2_ levels than the SCs under both untreated and early nematode infection conditions, and nematode exposure stimulated increased H_2_O_2_ production. These results further demonstrated that RCs had higher basal levels of H_2_O_2_ than SCs.

### 3.2. Differential Responses of H_2_O_2_-Related Enzyme Activity in RKN-Infected Sweetpotato Roots

**Figure 2:**
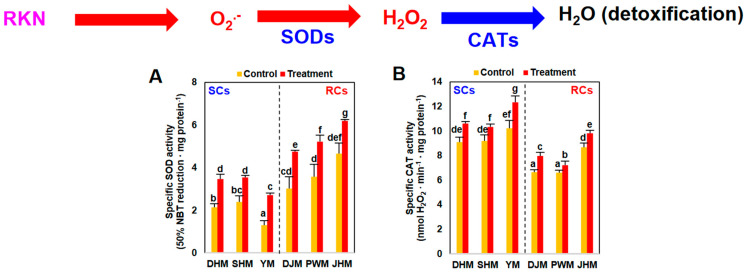
H_2_O_2_-related enzyme activities in sweetpotato cultivars 7 days after RKN infection. (**A**) H_2_O_2_-generating SOD activity and (**B**) H_2_O_2_-scavenging CAT activity in untreated and RKN-treated sweetpotato roots. Bars denoted with the same letter are not significantly different (*p* = 0.05) according to Tukey’s test. SCs, susceptible cultivars; RCs, resistant cultivars; DHM, Dahomi; SHM, Shinhwangmi; YM, Yulmi; DJM, Danjami; PWM, Pungwonmi; and JHM, Juhwangmi.

Changes in H_2_O_2_-related enzyme activities were investigated in RC and SC sweetpotato roots after RKN infection (Figure 2). Consistent with the elevated H_2_O_2_ levels in RCs and SCs (Figure 1B), the activity of H_2_O_2_-generating SOD increased after RKN infection in all six cultivars (Figure 2A). RCs had higher SOD activity levels than SCs. CAT, a representative antioxidant enzyme that detoxifies cells by removing H_2_O_2_, exhibited small changes in activity after infection (Figure 2B). In contrast with SOD activity, SCs had higher CAT activities than RCs in both treated and untreated conditions. Therefore, RCs exhibited higher H_2_O_2_-generating SOD activities and lower H_2_O_2_-removing CAT activities than SCs.

### 3.3. Differential Responses of Phenylpropanoid and Lignin Metabolism in RKN-Infected Sweetpotato Roots

**Figure 3:**
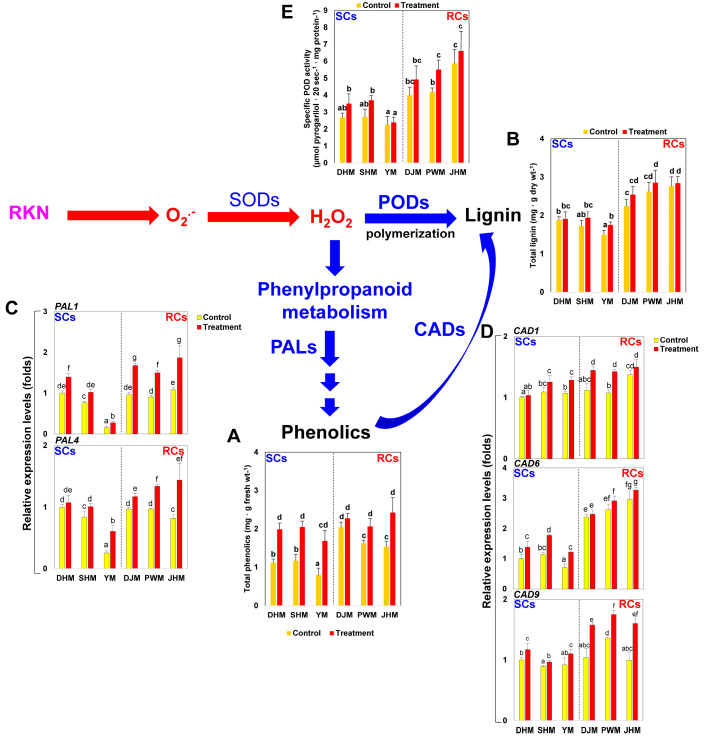
Phenylpropanoid metabolism-related activities in sweetpotato roots 7 days after RKN infection. (**A**) Total phenolic content. (**B**) Total lignin content. (**C**) Expression of *PAL* genes. (**D**) Expression of *CAD* genes. (**E**) POD enzyme activity. Bars denoted with the same letter are not significantly different (*p* = 0.05) according to Tukey’s test. SCs, susceptible cultivars; RCs, resistant cultivars; DHM, Dahomi; SHM, Shinhwangmi; YM, Yulmi; DJM, Danjami; PWM, Pungwonmi; and JHM, Juhwangmi.

During RKN infection, plants exhibit changes in their secondary metabolic pathways, including in the phenylpropanoid and lignin biosynthetic pathways. In this study, changes in the levels of total phenolics and lignin, which are intermediate products of the phenylpropanoid biosynthetic pathway, were investigated in sweetpotato roots during RKN infection. Total phenolic contents were higher in RCs than in SCs in the absence of infection. After RKN treatment, total phenolic contents increased in both SCs and RCs, but the increase was greater in RCs (Figure 3A). Total lignin contents did not increase significantly after RKN treatment compared with untreated control conditions for both SCs and RCs. As with total phenolic content, the total lignin content was higher in the three RCs than in the three SCs (Figure 3B). The expression levels of two phenylalanine ammonia-lyase genes (*PAL1* and *PAL4*) involved in regulating the phenylpropanoid biosynthetic pathway were higher in RKN-treated RCs than in SCs, similar to the total phenol content changes (Figure 3C). In addition, cinnamyl alcohol dehydrase (*CAD*) genes involved in monolignol biosynthesis, the final step of the lignin biosynthesis pathway, showed higher expression levels in RCs than in SCs, consistent with total lignin contents (Figure 3D). Guaiacol peroxidase (POD) removes H_2_O_2_ using various substrates, including phenolic compounds. During this process, monolignol is used as a substrate and is polymerized to form lignin polymers [11]. In this study, higher POD activities were observed in RCs than in SCs during RKN infection, suggesting that POD activity is likely involved in lignin accumulation (Figure 3E).

### 3.4. Changes in H_2_O_2_ Levels and Antioxidant Enzyme Activities during RKN Infection

**Figure 4:**
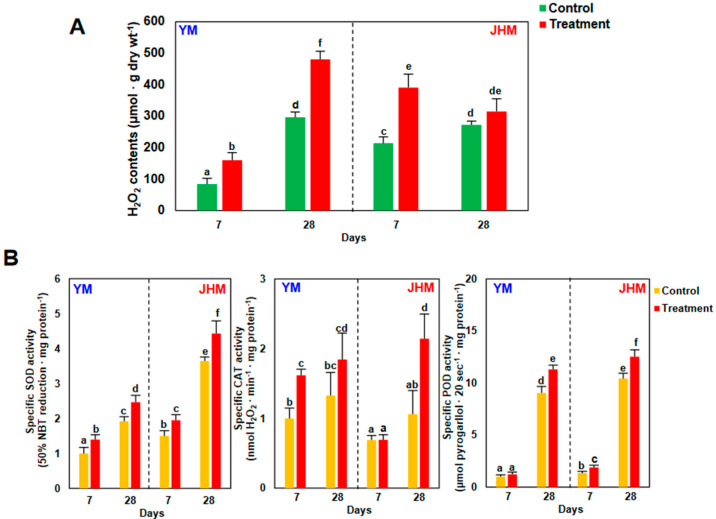
H_2_O_2_ contents and H_2_O_2_-related antioxidant enzyme activities in sweetpotato roots 7 days and 4 weeks after RKN infection. (**A**) H_2_O_2_ contents. (**B**) SOD, CAT, and POD enzyme activities. Bars denoted with the same letter are not significantly different (*p* = 0.05) according to Tukey’s test. SCs, susceptible cultivars; RCs, resistant cultivars; DHM, Dahomi; SHM, Shinhwangmi; YM, Yulmi; DJM, Danjami; PWM, Pungwonmi; and JHM, Juhwangmi.

Changes in H_2_O_2_-related responses were examined during early (7 days) and late (28 days) RKN infection periods by assessing H_2_O_2_ levels and antioxidant enzyme activities in a representative RKN-susceptible cultivar, Yulmi (YM), and a representative RKN-resistant cultivar, Juhwangmi (JHM). In the susceptible cultivar (YM), H_2_O_2_ levels increased over time in both RKN-treated and untreated roots, and after 28 days, H_2_O_2_ levels were higher in infected roots than in control roots (Figure 4A). In the resistant cultivar (JHM), the H_2_O_2_ levels were significantly higher in infected roots than in uninfected roots after 7 days, but there was no difference in the H_2_O_2_ levels between treated and untreated roots after 28 days. The activities of H_2_O_2_-related antioxidant enzymes SOD, CAT, and POD were analyzed in both YM and JHM at 7 and 28 days after RKN infection (Figure 4B). SOD activity was generally higher in JHM than in YM, and activity was significantly elevated in both cultivars 7 and 28 days after RKN infection. CAT activity increased upon RKN infection in YM after 7 and 28 days, but the difference was significant only at 7 days. No difference in CAT activity was seen between infected and uninfected JHM roots 7 days after treatment, but CAT activity was significantly higher in infected roots 28 days after treatment. POD activity increased substantially from 7 to 28 days in both cultivars, regardless of infection, and infected roots had significantly higher POD activities than control roots 28 days after infection.

## 4. Discussion

ROS play an important role in plant defense. During pathogen attack, levels of ROS-detoxifying enzymes such as APX and CAT are often suppressed in resistant plants [33]. As a result, plants produce more ROS, and the accumulation of these components leads to HR in plant cells. For example, H_2_O_2_ is involved in triggering HR in incompatible interactions [34]. In incompatible interactions, ROS are usually generated in a two-phase manner, with an initial rapid accumulation of ROS followed by a more stable second burst [35]. This response, which occurs several days after induction, appears to be related to plant innate immunity and is driven by changes in membrane potential, ion flux, and ROS production [36]. It is now well established that parasite and/or wound recognition and regulation of ROS contribute to the activation of plant defense responses [33,37]. However, it cannot be concluded that the resistance response to nematode infection is necessarily related to HR accumulation. Non-HR-mediated resistance responses were observed in Hsp1pr1-mediated resistance of sugar beet to the CN *Heterodera schachtii*, where J2 died due to degradation of the feeding structure [38]. Delayed HR-mediated cell death was also observed in the Hero-mediated genetic responses of tomato to the cyst nematodes *Globodera pallida* and *G. rostochiensis* via HR-mediated cell death in the developed syncytium [39]. Additionally, in the cowpea–RKN incompatibility interaction mediated by the *Rk* gene, classical HR characteristics of gene-to-gene resistance pathways were not observed [40]. ROS quantification results in RKN-infected cowpea roots showed that there was an early oxidative burst in both compatible and incompatible interactions in susceptible and resistant roots, respectively, but no significant difference between resistant and susceptible plants in ROS levels. In addition, no biphasic pattern of ROS generation was observed in incompatible reactions in cowpea roots. These results indicate that the initial bursts detected in cowpea roots upon RKN infection form part of the basal host defense response and are independent of genetic *Rk*-mediated resistance. The magnitude of H_2_O_2_ production was not sufficient to trigger HR cell death in cowpea roots. It is also possible that ROS scavenging mechanisms were not inhibited to a level sufficient to allow ROS to diffuse into cells to trigger HR. Therefore, the levels and timing of ROS produced during nematode infection can have variable consequences on nematode resistance response in a range of nematode–plant interactions.

In this study, the roots of RC sweetpotato had higher H_2_O_2_ levels than SC cultivars under non-infected conditions, and H_2_O_2_ levels increased strongly in RCs during the initial nematode infection period up to the 7th day after treatment (Figure 1). By contrast, the H_2_O_2_ level increased in the SC YM by day 28 after treatment, i.e., during the late infection stage (Figure 4). Previous transcriptome analysis of RCs and SCs showed a higher constitutive expression of resistance genes in RCs than SCs under normal conditions, and an increase in resistance gene expression was confirmed after infection [23]. This was consistent with the results in this study, where activated H_2_O_2_ and ROS defense responses were maintained at high levels in uninfected RCs and also increased rapidly after nematode infection. Conversely, nematode SCs showed lower H_2_O_2_ levels than nematode RCs under non-infected conditions, and levels only increased during the late infection stage. Therefore, nematode RCs displayed higher levels of H_2_O_2_ and ROS metabolism under non-infected conditions through constitutive expression of defensive genes, allowing a rapid response upon nematode infection. SCs with relatively low constitutive H_2_O_2_ levels and ROS metabolism are thought to initiate a defense against nematode infection only after infection occurs, resulting in increased levels only at a relatively late stage of infection.

One of the major aspects of ROS metabolism in plant cells is the generation and scavenging of H_2_O_2_ through SOD and CAT. This activation and inactivation of H_2_O_2_ regulates signal transduction and governs plant responses to pathogen infection [41,42]. Upon RKN infection, SOD and CAT responses led to the initiation of H_2_O_2_-mediated plant defenses. Interestingly, significant differences in enzyme activities were observed between RCs and SCs during the early phase of RKN infection. Increases in SOD and CAT activity in SCs reflected weak increases in H_2_O_2_ levels during RKN infection, whereas increases in SOD activity alongside decreases in CAT activity early in RKN infection in RCs resulted in higher increases in H_2_O_2_ levels.

Interactions between the H_2_O_2_ and phenylpropanoid biosynthetic pathways were also examined. Melillo et al. [12] demonstrated that RKN infection stimulated an increase in H_2_O_2_ production in tomato roots and activated the HR response in RKN-resistant tomatoes. Transcriptome analysis of RKN infection in tomato roots showed that RKN-induced changes in the flavonoid biosynthetic and lignin biosynthetic pathways were lower in RKN-susceptible tomatoes and highlighted the involvement of hormone signaling pathways in resistance and susceptibility to RKNs [43]. In this study, RKN infection led to increases in the total contents of phenolic and lignin compounds, which are phenylpropanoid-related metabolites, at higher levels in RCs than in SCs (Figure 3). Changes in POD activities were also observed during RKN infection, suggesting that oxidative polymerization through POD might be responsible for the increase in lignin content. These data indicate that higher H_2_O_2_ levels in RKN-infected sweetpotato roots during the early infection phase may be involved in resistance mechanisms, including the phenylpropanoid biosynthetic pathway.

**Figure 5:**
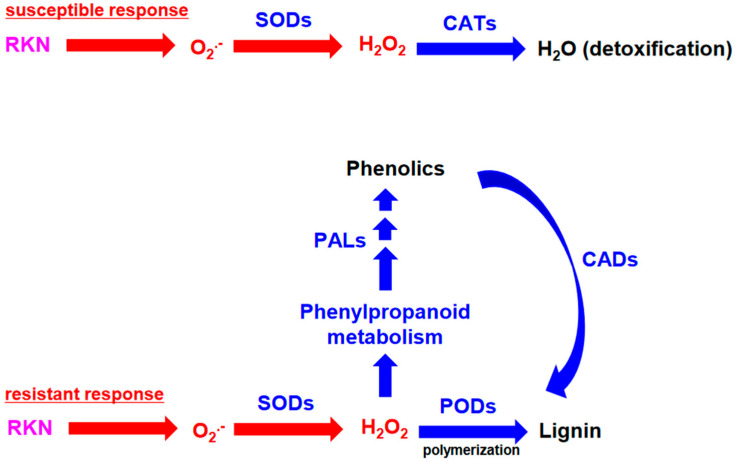
Proposed model of H_2_O_2_-related biological processes and genes influencing the RKN response in sweetpotato roots during early stages of infection. In susceptible cultivars, RKN infection induces SOD and CAT enzyme activities to scavenge and detoxify H_2_O_2_. In resistant cultivars, the induction of SOD activity and the repression of CAT activity increase H_2_O_2_ levels and may enhance phenylpropanoid metabolism-related defenses. Increased expression of genes encoding phenylpropanoid biosynthesis pathway factors, such as *PAL* genes, leads to an increase in total phenolics upon exposure to RKN infection in sweetpotato roots. CADs and PODs are also potentially involved in lignin accumulation-mediated responses during the early phase of RKN infection. RKN, root-knot nematode; SOD, superoxide dismutase; CAT, catalase; POD, peroxidase; PAL, phenylalanine ammonia-lyase; and CAD, cinnamyl alcohol dehydrogenase.

This study addressed the susceptibility and resistance responses of sweetpotato during RKN infection, focusing on changes in ROS metabolism, and showed how the production and transfer of H_2_O_2_ correlated with cultivars that were susceptible or resistant to RKNs (Figure 5). After RKN infection in SCs, SOD-mediated increases in H_2_O_2_ levels were accompanied by the activation of CAT, which detoxified H_2_O_2_ and reduced the overall H_2_O_2_ levels. By contrast, CAT was not activated to the same extent in RCs, leading to higher H_2_O_2_ accumulation and the activation of the phenylpropanoid and POD-mediated lignin pathways. These results suggest that increases in gene expression and enzyme activities of H_2_O_2_ metabolic components during RKN infection play an important role in enabling the resistance of sweetpotato to RKN infection.

## 5. Conclusions

This study examined the impact of H_2_O_2_ metabolic components on RKN resistance in sweetpotato. Differences in the expression of RKN-response-related genes and antioxidant enzyme activities were characterized in RKN-resistant and -susceptible sweetpotato cultivars. In susceptible sweetpotato cultivars, the SOD-mediated increase in H_2_O_2_ levels 7 days after RKN infection was accompanied by the activation of CAT, which detoxifies H_2_O_2_ and reduces total H_2_O_2_ levels. These changes were accompanied by low levels of POD activity, phenolic compounds, and lignin during the early response period, and increased H_2_O_2_ levels during the late response 28 days after RKN infection. By contrast, higher H_2_O_2_ accumulation occurred earlier in RCs, within the first 7 days after RKN infection, than in SCs. This led to the activation of the phenylpropanoid- and POD-mediated lignin pathway in RCs, and lower levels of H_2_O_2_ by 28 days after RKN infection. These results suggest that the increase in gene expression and enzyme activity of H_2_O_2_ metabolic components during the first 7 days after RKN infection play an important role in enabling the resistance of sweetpotato to RKN infection. This experimental approach identified differences in candidate gene expression and enzyme activities associated with changes in specific responses related to H_2_O_2_-related metabolic pathways. The development of transgenic lines exhibiting alterations in relevant gene expression or enzyme activities will allow further analysis of their impact on RKN resistance in sweetpotato. These results and lines of inquiry are valuable for the future development of crops with improved resistance to RKN infection through the regulation of H_2_O_2_ metabolism.

## Data Availability

Data are contained within the article.

## Supplementary Materials

The following supporting information can be downloaded at: https://www.mdpi.com/article/10.3390/antiox12061164/s1.

## Abbreviations

CAD	cinnamyl alcohol dehydrogenase
CAT	catalase
DHM	Dahomi
DJM	Danjami
JHM	Juhwanhmi
PAL	phenylalanine ammonia-lyase
POD	guaiacol peroxidase
PWM	Pungwonmi
RCs	resistant cultivars
RKN	root-knot nematode
SCs	susceptible cultivars
SHM	Shinhwangmi
SOD	superoxide dismutase
YM	Yulmi
